# Neuroprotective Effects of B-Type Cinnamon Procyanidin Oligomers on MPP^+^-Induced Apoptosis in a Cell Culture Model of Parkinson’s Disease

**DOI:** 10.3390/molecules26216422

**Published:** 2021-10-24

**Authors:** Qi Xu, Ziyu Chen, Borong Zhu, Yiming Li, Manju B. Reddy, Huilin Liu, Guodong Dang, Qi Jia, Xiaojun Wu

**Affiliations:** 1 School of Public Health, Shanghai University of Traditional Chinese Medicine, 1200 Cailun Road, Shanghai 201203, China; isuxuqi@shutcm.edu.cn (Q.X.); lynnnn0303@163.com (H.L.); kyctcm@vip.sina.com (G.D.); 2 Shanghai Key Laboratory of Compound Chinese Medicine, The Ministry of Education (MOE) Key Laboratory for Standardization of Chinese Medicine, Institute of Chinese Materia Medica, Shanghai University of Traditional Chinese Medicine, 1200 Cailun Road, Shanghai 201203, China; czy714@163.com; 3 School of Pharmacy, Shanghai University of Traditional Chinese Medicine, 1200 Cailun Road, Shanghai 201203, China; zhuborong1993@163.com (B.Z.); ymlius@163.com (Y.L.); 4 Department of Food Science and Human Nutrition, Iowa State University, Ames, IA 50010, USA; mbreddy@iastate.edu

**Keywords:** Parkinson’s disease, CPO-B, MPP^+^, Erk1/2, neuroprotection

## Abstract

Cinnamon procyanidin oligomers (CPOs) are water-soluble components extracted from cinnamon. This study aims to explore the neuroprotection of B-type CPO (CPO-B) against 1-methyl-4-phenylpyridinium (MPP^+^)-mediated cytotoxicity and the molecular mechanisms underlying its protection. The results demonstrated that CPO-B showed protection by increasing cell viability, attenuating an intracellular level of reactive oxygen species, downregulating cleaved caspase-3 expression, and upregulating the Bcl-2/Bax ratio. Moreover, CPO-B completely blocked the dephosphorylation of extracellular, signal-regulated kinase 1 and 2 (Erk1/2) caused by MPP^+^. Treatment with an Erk1/2 inhibitor, SCH772984, significantly abolished the neuroprotection of CPO-B against MPP^+^. Taken together, we demonstrate that CPO-B from cinnamon bark provided protection against MPP^+^ in cultured SH-SY5Y cells, and the potential mechanisms may be attributed to its ability to modulate the dysregulation between pro-apoptotic and anti-apoptotic proteins through the Erk1/2 signaling pathway. Our findings suggest that the addition of cinnamon to food or supplements might benefit patients with PD.

## 1. Introduction

Parkinson’s disease (PD) is a devastating and irreversible degenerative disorder that afflicts about 1.5% of the population aged 65 years and over in the world [1]. The cardinal features of PD include tremors, rigidity, postural instability, and slowness of voluntary movement, which are the results of the premature death of dopaminergic neurons and the deficiency of dopamine thereafter in the nigrostriatal system. Mitogen-activated protein kinases (MAPKs) are a type of serine/threonine protein kinases, consisting of extracellular, signal-regulated kinase (Erk) 1 and 2 (Erk1/2), P38 isoforms, and c-Jun N-terminal kinase (JNK) [2]. They are essential components of the signaling network and function as an integral part in cellular processes, such as growth, differentiation, apoptosis, survival, and proliferation [3]. Although the exact mechanisms responsible for neuronal death in PD remain unclear, research suggests that abnormal regulation of MAPKs may contribute to the pathogenesis of PD by triggering the dysregulation of pro-apoptotic and anti-apoptotic pathways, inducing neuroinflammation, oxidative stress, and mitochondrial dysfunction [4,5]. Thus, MAPK signaling pathways are considered as a possible therapeutic solution to PD treatment.

1-Methyl-4-phenylpyridinium (MPP^+^) is the ultimate metabolite of 1-methyl-4-phenyl-1,2,3,6 tetrahydropyridine (MPTP), causing the degeneration of the nigrostriatal dopaminergic neurons. MPP^+^ has been used extensively in laboratories to establish in vitro models of PD. The accumulation of MPP^+^ in the mitochondria interferes with the mitochondrial respiratory chain complex I, reduces mitochondrial respiration, and stimulates the generation of reactive oxygen species (ROS) [6]. It is also reported that MPTP/MPP^+^-induced neurodegeneration is associated with the dysregulation of MAPK signaling cascades or other signaling pathways such as protein kinase B (AKT) both in cell culture and animal models of PD [7,8,9]. For example, it is demonstrated that MPTP/MPP^+^-mediated suppression of Akt and Erk1/2 signaling pathways can downregulate the level of B-cell lymphoma 2 (Bcl-2)/Bcl-2-associated X protein (Bax) and promote caspases activation, leading to neuronal apoptosis [2,7,10]. We used an SH-SY5Y cell line in our experiments. When treated with neurotoxicants, the neurodegeneration recapitulated in SH-SY5Y cells serves as a model for mechanisms of dopaminergic neuronal death observed in PD [11]. Moreover, we selected undifferentiated SH-SY5Y cells since research suggests that they are suitable for the neuroprotection studies, with high vulnerability to neurotoxins such as MPP^+^ [12].

Functional food has gained attention in the past few decades since people are not only concerned about the taste but also the nutritional values and health benefits. Cinnamon contains many functional food ingredients such as cinnamaldehyde, cinnamic acid, polyphenols, and flavonoids. Cinnamon is an important spice used by people worldwide and has both culinary as well as medicinal uses. The beneficial effects of cinnamon, as suggested by research, include its anti-inflammatory, antioxidant, and antidiabetic activities [13]. The extracts from cinnamon such as cinnamaldehyde and flavonoids were found to possess strong antioxidant and anti-inflammatory properties to combat chronic diseases. Cinnamon polyphenols were demonstrated to have insulin-like activities and regulate glucose metabolism [13]. Cinnamon extracts have also shown neuroprotective effects through upregulating the neurotrophic factors in PD and reducing the formation of toxic β amyloid polypeptides in Alzheimer’s disease [14,15]. Active water-soluble components of cinnamon were isolated and identified as cinnamon procyanidin oligomers (CPOs). There are two main types of CPOs in plants, namely, A- and B-types, based on the interflavan linkage between the flavan-3-ol units [16]. Our previous studies also confirmed the existence of both A- and B-types of CPOs from cinnamon bark [16,17].

The beneficial effects of procyanidins have gained attention, and it has been demonstrated that procyanidins extracted from blueberries and grape seeds may alleviate neurodegeneration via the improvement of antioxidant enzymes, the enhancement of mitochondrial function, and the regulation of MAPK signaling pathways [18,19]. However, the neuroprotective effects of procyanidins extracted from cinnamon are seldomly reported. CPO-B is the major procyanidin in *Cinnamomum cassia* (L.), which is the most popular cinnamon bark in China. However, its beneficial effects in PD are not well studied. This study aims to assess whether CPO-B attenuates MPP^+^-induced cytotoxicity in the dopaminergic cell SH-SY5Y, a cell model of PD, and to explore whether it exerts neuroprotection through activating the Erk1/2 signaling pathway and modulating the dysregulation between pro-apoptotic and anti-apoptotic proteins. These findings may have implications for the application of CPO-B in the therapy of PD.

## 2. Results

### 2.1. Cytotoxic Effects of MPP^+^ and CPO-B

The optimal dose of MPP^+^ as well as that of CPO-B used in later experiments was determined in a dose-response study of MPP^+^ and CPO-B on SH-SY5Y cells using the MTS assay (Table 1). The cells were treated with increasing concentrations of MPP^+^ or CPO-B for 12 h. As shown in Table 1, cell viability was reduced (*p* < 0.001) to 93%, 87%, 72%, 57%, 45%, and 34% with 0.1 mM, 0.5 mM, 1 mM, 2.5 mM, 5 mM, and 10 mM of MPP^+^, respectively. CPO-B did not show any significant cytotoxicity after the 12 h incubation with a concentration less than 50 µM. Based on these results, 1 mM MPP^+^ and 12.5 µM CPO-B were chosen to evaluate the neuroprotection of CPO-B in the following experiments.

### 2.2. CPO-B Protects SH-SY5Y Cells from MPP^+^-Induced Intracellular ROS Production and Apoptosis

**Figure 1:**
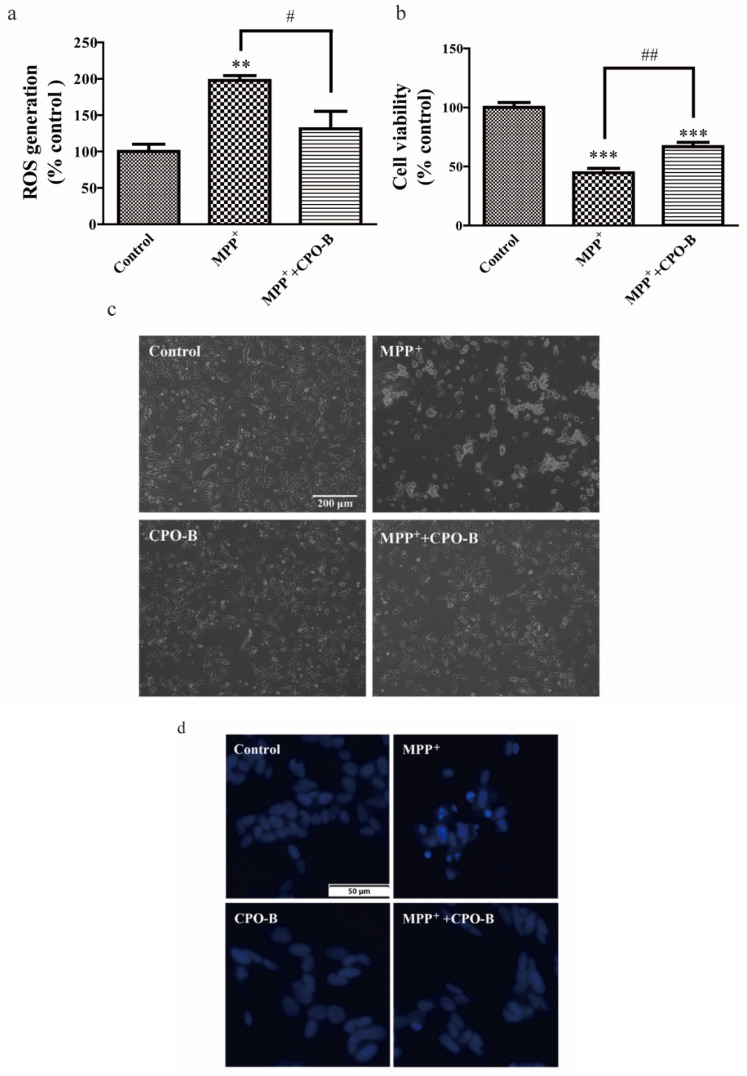
The effect of CPO-B on MPP^+^-induced cytotoxicity measured by intracellular ROS generation (**a**, *n* = 4), cell viability (**b**, *n* = 8)**,** microscopic examination (**c**) and Hoechst nuclear staining (**d**); the values (mean ± SEM) were standardized to the percentage of control; symbol * indicates the difference between control and other treatments; symbol # indicates the difference between MPP^+^ and MPP^+^+CPO-B; ** *p* < 0.01, *** *p* < 0.001, # *p* < 0.05, ## *p* < 0.01; CPO-B, B-type cinnamon procyanidin oligomer; MPP^+^, 1-methyl-4-phenylpyridinium.

In order to evaluate whether CPO-B suppressed MPP^+^-mediated intracellular ROS generation and apoptosis, cells were incubated with 12.5 µM CPO-B for 2 h, and 1 mM MPP^+^ for another 12 h. We found MPP^+^ treatment stimulated intracellular ROS production by twofold (*p* < 0.01) and decreased cell viability by 55% (*p* < 0.001) (Figure 1a,b). However, 12.5 μM CPO-B showed significant protection by reducing intracellular ROS by 34% (*p* < 0.05) and increasing cell viability by 49% (*p* < 0.01). In agreement with the above results, microscopic morphological examination also indicated the protective effects of CPO-B (Figure 1c). Cells without treatment or treated with CPO-B alone were round with regular homogeneous staining of Hoechst 33258. However, MPP^+^-induced apoptotic cells exhibited condensed and fragmented nuclei with a strong and bright florescence of Hoechst stain. CPO-B pretreatment markedly blocked the morphological damage caused by MPP^+^ and resulted in a non-apoptotic cell phenotype in the visual fields (Figure 1c,d).

### 2.3. CPO-B Blocks MPP^+^-Induced Alteration of Apoptosis-Associated Proteins in SH-SY5Y Cells

**Figure 2:**
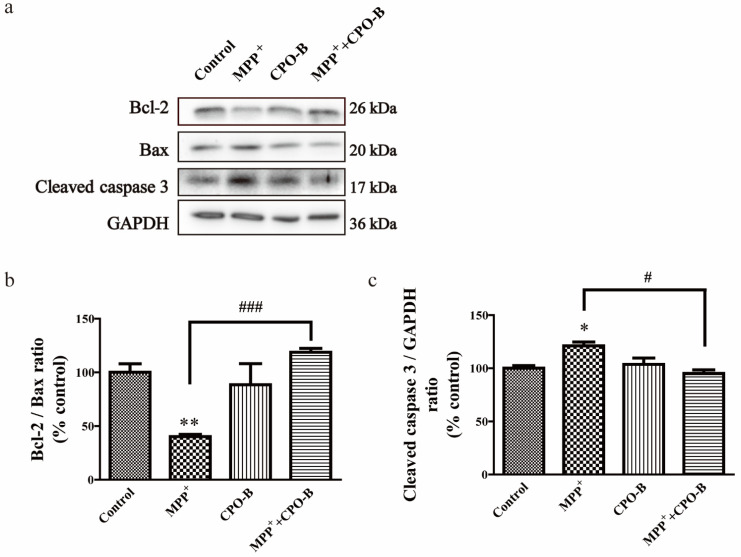
The effect of CPO-B on MPP^+^-induced alteration of Bcl-2/Bax expression (**b**, *n* = 3) and cleaved caspase-3 expression (**c**, *n* = 3). The top panel shows the representative Western blots (**a**); the values (mean ± SEM) were standardized to the percentage of control; symbol * indicates the difference between control and other treatments; symbol # indicates the difference between MPP^+^ and MPP^+^+CPO-B; * *p* < 0.05, ** *p* < 0.01, # *p* < 0.05, ### *p* < 0.001; CPO-B, B-type cinnamon procyanidin oligomer; MPP^+^, 1-methyl-4-phenylpyridinium; Bcl-2, B-cell lymphoma 2; Bax, Bcl-2-associated X protein; GAPDH, glyceraldehyde 3-phosphate dehydrogenase.

We further evaluated whether CPO-B provided protection against MPP^+^-induced apoptosis by examining the alteration of apoptosis-associated proteins, including cleaved caspase-3, Bcl-2, and Bax (Figure 2a). MPP^+^ downregulated the expression of Bcl-2 with no significant effects on the expression of Bax, resulting in a 60% decrease in the Bcl-2/Bax ratio when compared to control cells (*p* < 0.01) (Figure 2b). However, CPO-B significantly blocked the effects of MPP^+^ and increased the Bcl-2/Bax ratio to 78% of the control cells (*p* < 0.001), suggesting the anti-apoptotic role of CPO-B. Moreover, MPP^+^ also upregulated the expression of cleaved caspase-3 by 17% of the control (*p* < 0.05), and CPO-B pretreatment completely counteracted the effects (*p* < 0.05) and the expression of cleaved caspase-3 was downregulated to the control level (Figure 2c).

### 2.4. CPO-B Exerts Neuroprotection through the Upregulation of Phosphorylation of Erk1/2 but Not AKT Expression

**Figure 3:**
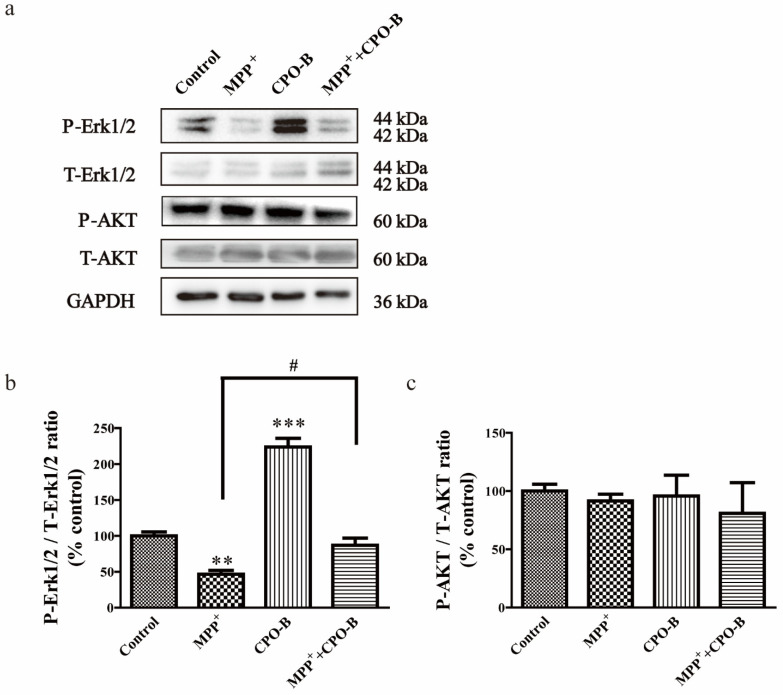
The effect of CPO-B on the MPP^+^-induced alteration of Erk1/2 (**b**, *n* = 3) and Akt phosphorylation (**c**, *n* = 3). The top panel in the figure shows representative Western blots (**a**). The values (mean ± SEM) were normalized to control; symbol * indicates the difference between control and other treatments; symbol # indicates the difference between MPP^+^ and MPP^+^+CPO-B; ** *p* < 0.01, *** *p* < 0.001, # *p* < 0.05; CPO-B, B-type cinnamon procyanidin oligomer; MPP^+^, 1-methyl-4-phenylpyridinium; AKT, protein kinase B; P-AKT, phosphorylated AKT; T-AKT, total AKT; Erk1/2, extracellular signal regulated kinase 1 and 2; P-Erk1/2, phosphorylated Erk1/2; T-Erk1/2, total Erk1/2; GAPDH, glyceraldehyde 3-phosphate dehydrogenase.

For the purpose of exploring the possible molecular mechanisms behind the protective effects of CPO-B, we further examined the protein expression of Erk1/2 and Akt (Figure 3a). MPP^+^ treatment significantly suppressed the expression of phosphorylated Erk1/2 (P-Erk1/2) but not total Erk1/2 (T-Erk1/2), resulting in a 53% decrease (*p* < 0.01) in the ratio of P-Erk1/2/T-Erk1/2 in comparison with the control (Figure 3b). CPO-B completely blocked the effects (*p* < 0.05) and upregulated the ratio of P-Erk1/2/T-Erk1/2 to the control level. However, neither MPP^+^ nor CPO-B treatment had a significant effect on Akt phosphorylation (Figure 3c).

**Figure 4:**
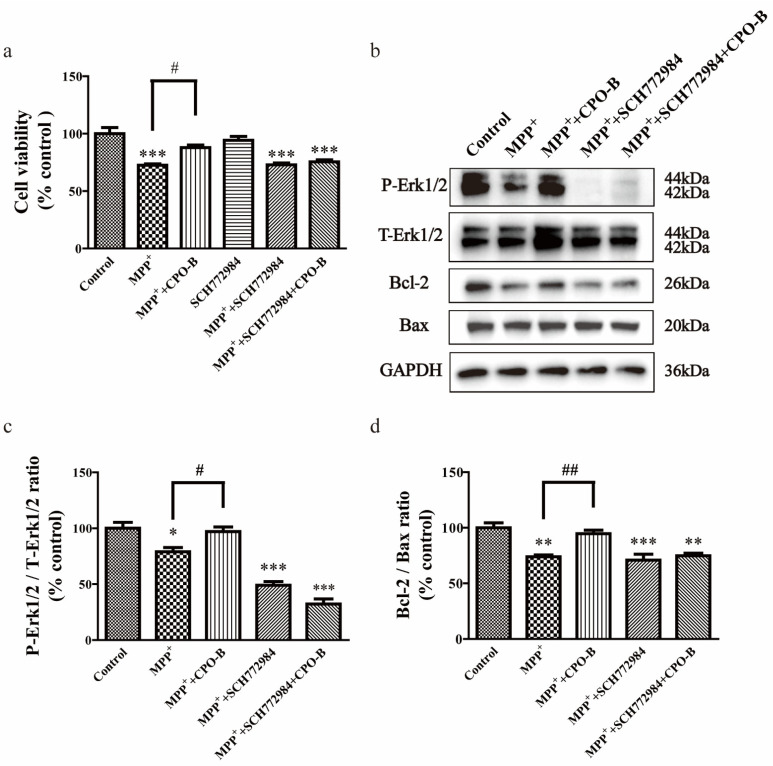
The effect of Erk1/2 inhibition on CPO-B-mediated neuroprotection measured by cell viability (**a**, *n* = 3), Erk1/2 phosphorylation (**c**, *n* = 3) and Bcl-2/Bax expression ratio (**d**, *n* = 3). The top panel in the figure shows representative Western blots (**b**). The values (mean ± SEM) were normalized to control; symbol * indicates the difference between control and other treatments; symbol # indicates the difference between MPP^+^ and MPP^+^+CPO-B; * *p* < 0.05, ** *p* < 0.01, *** *p* < 0.001, # *p* < 0.05, ## *p* < 0.01. CPO-B, B-type cinnamon procyanidin oligomer; MPP^+^, 1-methyl-4-phenylpyridinium; Bcl-2, B-cell lymphoma 2; Bax, Bcl-2-associated X protein; Erk1/2, extracellular signal-regulated kinase 1 and 2; P-Erk1/2, phosphorylated Erk1/2; T-Erk1/2, total Erk1/2; GAPDH, glyceraldehyde 3-phosphate dehydrogenase.

To further examine the role of the Erk1/2 pathway in CPO-B-mediated protection, the cells were treated with 2 µM Erk1/2 inhibitor SCH772984 for 2 h, followed by the treatment of CPO-B and MPP^+^. We observed that the SCH772984 treatment alone had no significant effects on cell viability (Figure 4a). However, the incubation of SCH772984 blocked the protective effects of CPO-B by inhibiting Erk1/2 phosphorylation and completely diminishing the effects of CPO-B on MPP^+^-induced downregulation of Bcl-2 (Figure 4b–d). These results demonstrated that the Erk1/2 pathway is indeed involved in CPO-B-mediated neuroprotection.

## 3. Discussion

PD is a neurodegenerative disease that affects around 1% of the population over the age of 60 and 4% of those over the age of 80 [4]. However, there is no known cure for PD, and current treatments only alleviate the symptoms. The major goal of PD research is to identify therapeutic agents that could either delay or stop the disease’s progression.

Food-derived phenolic compounds have gained particular attention as a promising therapeutic approach against neurodegenerative disorders in recent years. A number of polyphenol compounds, such as resveratrol from grape seeds or curcumin, as well as epigallocatechin gallate (EGCG) from tea, have been reported to exert neuroprotection through the scavenging of free radicals, suppressing mitochondrial dysfunction, and alleviating neuroinflammation [20,21]. Procyanidins are a class of polyphenols wildly present in many vegetables, fruits, nuts, and seeds, and are considered to be among the most potent antioxidants in nature. Research has demonstrated their beneficial effects, including reducing the risk of cerebrovascular disease, diabetes, cardiovascular disease, and cancer mortality [22]. One study also found that pretreatment with procyanidins could significantly alleviate rotenone-induced oxidative stress and reduce apoptosis in SH-SY5Y cells [19]. However, this study did not reveal where the procyanidins were extracted from, nor did it mention the degree of polymerization of the procyanidins. Since procyanidins are polymeric flavanols with a variety of linkages and subunits, the degree of polymerization and connectivity determines their bioavailability and bioactivity [23]. For example, research shows that B-type procyanidin polymers more effectively inhibited inflammation compared to monomers and oligomers in human colon cells [24]. Another study shows that the oligomeric form of procyanidins has a stronger protection ability in regard to reducing inflammation and oxidative stress than that of polymers in diabetic rats [25]. In our current study, CPO-B from cinnamon bark was shown to remarkably protect against MPP^+^-induced neurotoxicity in cultured SH-SY5Y cells, suggesting the possible application of CPO-B in the therapy of PD.

The neuroprotective effects of CPO-B in our study raise issues about its absorption and the capability to cross the blood–brain barrier. Research has demonstrated that procyanidin oligomers such as dimer and trimer procyanidins are absorbable in vivo and reach maximum concentrations one hour after digestion [26]. The major components of CPO-B include procyanidin B-2, procyanidin C-1, and cassiatannin A-2. One study found that these oligomeric procyanidins were absorbed by the small intestine and detected in the different tissues without structure modification after oral ingestion [27]. A human trial demonstrated that procyanidin B-2 could be absorbed by the human intestinal tract and detected in plasma [28]. The capabilities of procyanidin metabolites to cross the blood–brain barrier and to target the brain have also been observed in several in vivo studies [29,30]. Consistent with these findings, our recent study found that A-type CPO could cross the blood–brain barrier and provide neuroprotection in an animal model of PD [31].

Our current study found 1 mM MPP^+^ significantly downregulated Bcl-2, while Bax expression increased, and caspase-3 activity was activated, which was blocked by 12.5 µM CPO-B, suggesting an anti-apoptotic role of CPO-B. Although it is difficult to extrapolate the in vitro model to human physiological conditions, our study found no toxicity of CPO-B under the concentration of 50 µM. Moreover, based on the previous study showing that the absorption rate of the polyphenolic compound was 5–10% [32], we expected an adult with 5 L blood to take 500–1000 mg CPO-B daily to reach a concentration of 12.5 µM in their blood circulation. This is similar to the findings of our previous in vivo study estimating a daily dose of 720 mg for A-type CPO in the prevention of PD [31]. In addition, the data from NHANES 1999–2002 indicated that the total daily procyanidin intake in adults is around 95 mg, far below the dose used in our current study. However, our study still suggested the potential role of cinnamon procyanidin supplementation in the prevention of PD.

We further investigated the mechanisms underlying the neuroprotection of CPO-B against MPP^+^-induced apoptosis by examining the role of Erk1/2 and Akt kinases. Erk1/2 is one of the major signaling cassettes of the MAPK signaling pathway and plays an essential role during cell proliferation, death, differentiation, and survival [7]. Although Erk1/2 usually functions as a mediator against apoptosis, it is also found that Erk1/2 signaling can be pro-apoptotic depending on the cell scenario and the type of cell insults. For example, one study reported that Erk1/2 is pro-apoptotic in rotenone-induced cell death, and procyanidins exerted its neuroprotection by inhibiting Erk1/2 phosphorylation. However, our study found MPP^+^ reduced the phosphorylation of Erk1/2, suggesting that inhibition of Erk1/2 is involved in MPP^+^-induced cell death. Moreover, CPO-B significantly blocked MPP^+^-induced Erk1/2 dephosphorylation, and the Erk1/2 inhibitor SCH772984 diminished the protective effects of CPO-B. These results suggest that CPO-B exerted neuroprotection by increasing Bcl2 expression and inhibiting caspase 3 activation through Erk1/2 activation. Since Erk1/2 regulates the functions of various substrates in cells and activates multiple transcription factors associated with neuroinflammation and oxidative stress, future studies are needed to determine whether CPO-B exerts antioxidant and anti-inflammatory effects through Erk1/2 signaling pathways in experimental models of PD.

In our current study, the AKT signaling pathway was also examined, since it modulates various cellular functions, such as neuronal cell migration, proliferation, and plasticity, and provides an important signaling for neuroprotection [5]. Although studies have shown that dysregulation of AKT facilitated mitochondrial failure and contributed to MPP^+^-induced cell death [33,34], we did not observe significant changes in AKT activation following treatment with MPP^+^ or CPO-B. The different results may be due to the varied microenvironmental system in the experiments.

In conclusion, we extracted CPO-B from cinnamon and demonstrated that it exerted significant neuroprotection against MPP^+^-induced cytotoxicity. The underlying mechanisms included its ability of activating Erk1/2 phosphorylation, enhancing the ratio of Bcl-2/Bax expression and subsequently inhibiting caspase-3 activity. While the current study focused on the effects of CPO-B in mitigating the dysregulation of cellular survival signals induced by MPP^+^, future studies are underway to investigate the effects of CPO-B on dopamine system dysfunction in animal models of PD. The present findings not only provide pharmacological and mechanistic evidence to support potential therapeutic application of procyanidins in PD but also indicate that the addition of cinnamon to foods or using CPO-B as a dietary supplement might benefit patients with PD.

## 4. Materials and Methods

### 4.1. Chemicals

The SH-SY5Y cell line was supplied from Laboratory of Stem Cell Biology of Chinese Academy of Sciences (Shanghai, China). Hoechst 33258, streptomycin, penicillin, Dulbecco’s modified Eagle medium (DMEM), fetal bovine serum, and 0.25% trypsin/EDTA were acquired from Thermo Fisher (Carlsbad, CA, USA). The primary antibodies against glyceraldehyde 3-phosphate dehydrogenase (GAPDH), cleaved Caspase 3, Bax, Bcl-2, Akt, Erk1/2, phospho-Akt (Thr309/Thr308/Thr305), and phospho-Erk1/2 (Thr202/Tyr204, Thr185/Tyr187) were bought from Cell Signaling Technology (Danvers, MA, USA). MPP^+^ iodide was supplied from Sigma-Aldrich (St. Louis, Missouri, MO, USA). Erk1/2 inhibitor SCH772984 was purchased from Meilun (Dalian, China). One solution cell proliferation assay (MTS) was acquired from Promega (Madison, WI, USA). Before each assay, all solutions were freshly prepared.

### 4.2. The Extraction of Procyanidin

As described previously, CPO-B was isolated from *cassia bark* and subjected to macro-resin column chromatography eluted with water and a series of different concentrations of ethanol for purification [35]. The total percentage of CPO-B was nearly 60% and identified as cassiatannin A-2 (14.8%), procyanidin C-1 (18.7%), and procyanidin B-2 (26.1%), which was confirmed by high-performance liquid chromatography.

### 4.3. Design of Experiments

SH-SY5Y cells at a density of 1 × 10^6^/mL were grown in 96-well plates with DMEM medium with the supplementation of 10% fetal bovine serum and 1% penicillin/streptomycin in a humid chamber at 37 °C. The cells were treated with six concentrations of MPP^+^ (0.1 mM, 0.5 mM, 1 mM, 2.5 mM, 5 mM, 10 mM) and four concentrations of CPO-B (5 μM, 10 μM, 25 μM, 50 μM) to select the desired concentration of MPP^+^ and CPO-B for the subsequent experiments. To examine whether CPO-B provided protection against MPP^+^-induced toxicity, cells were treated with 1 mM MPP^+^ for 12 h with or without the pretreatment of CPO-B for 2 h. To further explore the role of Erk1/2 activation in CPO-B mediated neuroprotection, cells were pretreated with Erk1/2 inhibitor SCH772984 for 2 h followed by MPP^+^ with or without CPO-B treatments.

### 4.4. Cell Viability

An MTS assay was conducted to evaluate cell viability following the manufacturer’s recommendations. Following the treatment, the cells were exposed with 20 μL MTS reagent at 37 °C for 4 h in 96-well plates. The tetrazolium reagents were reduced by the cellular hydrogenases to yield the soluble formazan, which was quantified by Thermo Fisher Scientific Varioskan Flash at 490 nm (Waltham, MA, USA).

### 4.5. Measurement of Intracellular ROS

The intracellular ROS level was assessed by the cell permeable ROS sensor 5-(and-6)-chloromethyl-2′,7′-dichlorodihydrofluorescein diacetate (CM-H_2_DCFDA). After treatments, cells were loaded with 100 µL PBS containing 10 µM CM-H_2_DCFDA for 30 min in the dark. The fluorescence was quantified with Thermo Fisher Scientific Varioskan Flash (Waltham, MA, USA) at 490 nm excitation/530 nm emission.

### 4.6. Nuclear Staining with Hoechst 33258

Hoechst 33258 dye has been used extensively for the qualitative identification of nuclear apoptotic morphology [36]. Cells were plated on coverslips for 24 h before the treatments, fixed with 4% paraformaldehyde, and counterstained with 10 μg/mL Hoechst dye 33258.

### 4.7. Western Blotting Analysis

After the experimental treatments, cell pellets were lysed with a CelLytic^TM^ MT cell lysis buffer. The cell lysates were analyzed by SDS-polyacrylamide gel electrophoresis (10% or 12%) and blotted onto polyvinylidene fluoride membranes. The proper primary antibodies at 1/1000 dilution and the secondary antibodies at 1/5000 dilution were used following the manufacturer’s recommendations. GAPDH at 1/5000 dilution was used as an internal reference for an equal loading of protein.

### 4.8. Statistical Analysis

The values were standardized to the percentage of control and expressed as mean ± SEM in each experiment. The data were analyzed with a one-way ANOVA test with Tukey’s or Dunnett’s analysis using the GraphPad Prism 5 package (Graph Software, San Diego, CA, USA). The differences among treatments were considered statistically significant at *p* < 0.05.

## 5. Conclusions

In conclusion, we extracted CPO-B from cinnamon and demonstrated that it exerted significant neuroprotection against MPP^+^-induced cytotoxicity. The underlying mechanisms included its ability to activate Erk1/2 phosphorylation, enhancing the ratio of Bcl-2/Bax expression and subsequently inhibiting caspase-3 activity. While the current study focused on the effects of CPO-B in mitigating the dysregulation of cellular survival signals induced by MPP^+^, future studies are underway to investigate the effects of CPO-B on dopamine system dysfunction in animal models of PD. The present findings not only provide pharmacological and mechanistic evidence to support potential therapeutic application of procyanidins in PD but also indicate that the addition of cinnamon to foods or using CPO-B as a dietary supplement might benefit patients with PD.

## Figures and Tables

**Table 1:** The cytotoxic effects of MPP^+^ (*n* = 3) and CPO-B (*n* = 6) on SHSY-5Y cells by MTS assay.

Dose	Cell Viability (%)
MPP^+^ (mM)	
0	100 ± 0.6
0.1	93 ± 1.4 ***
0.5	87 ± 1.3 ***
1	72 ± 2.5 ***
2.5	57 ± 1.7 ***
5	45 ± 1.9 ***
10	34 ± 0.2 ***
CPO-B (µM)	
0	100 ± 2.0
5	94 ± 4.8
10	91 ± 6.1
25	83 ± 2.9
50	70 ± 2.5 ***

The values (mean ± SEM) were standardized to the percentage of control; symbol * indicates the difference between control and other treatments; *** *p* < 0.001.

## Data Availability

All data used to support the findings of this study are included within the article and they are also available from the corresponding author upon request.
